# ATG-Fresenius increases the risk of red blood cell transfusion after kidney transplantation

**DOI:** 10.3389/fimmu.2022.1045580

**Published:** 2022-12-01

**Authors:** Maria Sebti, Camille Petit-Hoang, Btissam Chami, Étienne Audureau, Catherine Cordonnier-Jourdin, Muriel Paul, Franck Pourcine, Philippe Grimbert, Clément Ourghanlian, Marie Matignon

**Affiliations:** ^1^ Pharmacy Department, Hôpitaux Universitaires Henri Mondor-Albert Chenevier, Assistance Publique-Hôpitaux de Paris (AP-HP), Créteil, France; ^2^ Nephrology and Renal Transplantation Department, Hôpitaux Universitaires Henri Mondor-Albert Chenevier, Assistance Publique-Hôpitaux de Paris (AP-HP), Créteil, France; ^3^ Etablissement Français du Sang (EFS) - Ile de France, Créteil, France; ^4^ Public Health Department, Hôpitaux Universitaires Henri Mondor-Albert Chenevier, Assistance Publique-Hôpitaux de Paris (AP-HP), Créteil, France; ^5^ AP-HP (Assistance Publique-Hôpitaux de Paris), Hôpitaux Universitaires Henri Mondor, Fédération Hospitalo-Universitaire TRUE (InnovaTive theRapy for immUne disordErs), Créteil, France; ^6^ Université Paris-Est Créteil, Institut National de la Santé et de la Recherche Médicale (INSERM) U955, Institut Mondor de Recherche Biomédicale (IMRB), Créteil, France; ^7^ AP-HP (Assistance Publique-Hôpitaux de Paris), Hôpitaux Universitaires Henri Mondor, CIC biotherapy, Créteil, France; ^8^ Prevention, Diagnosis and Treatment of Infections Department, Unité Transversale de Traitement des Infections, Hôpitaux Universitaires Henri Mondor-Albert Chenevier, Assistance Publique-Hôpitaux de Paris (AP-HP), Créteil, France

**Keywords:** kidney transplantation, anti-thymocyte globulins, donor-specific anti-HLA antibodies, red blood cell transfusion, induction therapy

## Abstract

**Introduction:**

In sensitized deceased donor kidney allograft recipients, the most frequent induction therapy is anti-thymocyte globulins (ATG), including Thymoglobulin® (Thymo) and ATG-Fresenius (ATG-F).

**Methods:**

We conducted a 3-year monocentric observational study to compare the impact of ATGs on hematological parameters. We included adult kidney transplant recipients treated with ATG induction therapy, either Thymo or ATG-F, on a one-in-two basis. The primary endpoint was red blood cell (RBC) transfusions within 14 days after transplantation.

**Results:**

Among 309 kidney allograft recipients, 177 (57.2%) received ATG induction, 90 (50.8 %) ATG-F, and 87 (49.2%) Thymo. The ATG-F group received significantly more RBC transfusions (63.3% vs. 46% p = 0.02) and in bigger volumes (p = 0.01). Platelet transfusion was similar in both groups. Within 14 and 30 days after transplantation, older age, ATG-F induction, and early surgical complication were independently associated with RBC transfusion. Patient survival rate was 95%, and the death-censored kidney allograft survival rate was 91.5% at 12 months post-transplantation. There was no difference in the incidence of acute rejection and infections or in the prevalence of anti-HLA donor-specific antibodies.

**Discussion:**

In conclusion, after kidney transplantation, ATG-F is an independent risk factor for early RBC transfusion and early thrombocytopenia without clinical and biological consequences. These new data should be clinically considered, and alternatives to ATG should be further explored.

## Introduction

Kidney transplantation remains to be the gold standard treatment of end-stage renal disease (ESRD) ensuring significant improvements in patient survival and quality of life (1, 2). Induction immunosuppressive treatment after kidney transplantation, including T-cell– or non-T-cell–depleting therapy, reduces the incidence and severity of acute rejection, delays the initiation of calcineurin inhibitors, and/or helps keep maintenance corticosteroids or calcineurin inhibitor therapy to the minimum (3). In highly sensitized deceased kidney allograft recipients, anti-thymocyte globulins (ATGs) are the most frequent induction treatment used as they immediately deplete immune T-cells, thus reducing the incidence of acute rejection or delayed graft function and improving kidney allograft survival better than non-T-cell–depleting monoclonal interleukin-2 receptor antagonists (3, 4). In living kidney allograft recipients, rituximab, plasma exchange, and donor-specific immunosuppression could replace ATGs with better results (5). ATG is polyclonal and therefore has diverse effects on the immune system: (i) T-cell depletion in blood and peripheral lymphoid tissues (main effect) through complement-dependent lysis, T-cell activation, and apoptosis; (ii) modulation of key cell surface molecules that mediate leukocyte/endothelium interactions; (iii) induction of apoptosis in B-cell lineages, i.e., interfering with dendritic cell functional properties; and (iv) induction of regulatory T and natural killer T cells (6, 7).

Currently, two ATGs are widely used: Thymoglobulin^®^ (Thymo) and ATG-Fresenius (ATG-F) (8). Both are rabbit-derived antibodies. ATG-Fresenius comprises only rabbit antibodies formed upon exposure to the Jurkat cell line, which resembles activated T cells (6). Thymoglobulin is a pasteurized antibody produced by immunizing pathogen-free New Zealand rabbits with fresh human thymocytes (6). For such, these two drugs target different antigen specificities, which could explain their different clinical effects. A recent meta-analysis reported better short-term kidney transplantation outcomes in patients treated with ATG-F as compared with those treated with Thymo, despite a significantly higher acute rejection rate in ATG-F-treated kidney transplant recipients (8, 9).

The safety profile covers hematological abnormalities, infections, and acute infusion-associated reactions (4). The specificity of ATG for T cell is not optimum, thus it is common to detect thrombocytopenia or anemia within days after injection (10–12). Thymo seems to induce more lymphopenia than ATG-F (9), whereas the latter causes significantly more post-transplantation thrombocytopenia (13). On the other hand, anemia and post-transplantation thrombocytopenia may necessitate blood product transfusion, a well-known factor of sensitization and development of anti-human leucocyte antigen (HLA) donor-specific antibodies (DSA) (14). After kidney transplantation, the emergence of DSA is often associated with antibody-mediated rejection (ABMR) and lower kidney allograft survival (15).

Several studies have compared the effectiveness of Thymo and ATG-F in kidney transplant recipients (9, 10, 16, 17) and recorded the effects of each ATG type on the blood (10–13). However, the studied immunosuppressive therapy was not always the same; the clinical consequences, including red blood cell (RBC) transfusions, were not analyzed in all studies; and no data on DSA developed after transplantation, especially after RBC transfusion, were available. We conducted a monocentric observational study to determine whether the use of Thymo or ATG-F increases the need for blood product transfusion in the first month after kidney allograft transplantation and to evaluate their clinical effects in the first post-transplantation year.

## Patients and methods

### Study design and population

Our study is a single-center observational study carried out at Henri Mondor Hospital, Creteil, France. All adult kidney recipients (> 18 years) transplanted between January 2016 and August 2018 and treated with anti-thymocyte globulin as induction therapy were included (have calculated panel reactive antibodies (cPRA) > 50%, second or more transplantation, and preformed anti-HLA donor-specific antibodies). We excluded multiorgan recipients. The local ethical committee authorized the study (IRB #00003835).

### Collected data

We collected demographic (age, sex, body mass index (BMI), comorbidities, dialysis), biological (blood cell count, serum creatinine, DSA), and therapeutic (erythropoietin, anticoagulation, antiplatelet) data.

Expanded criteria donor (ECD) was defined as donors older than 60 years or between 50 and 60 years, having two of the three following criteria: (i) hypertension; (ii) preretrieval serum creatinine > 1.50 mg/dl; and (iii) cerebrovascular cause of brain death (18). The glomerular filtration rate was estimated (eGFR) using the MDRD formula (19). We identified delayed graft function (DGF) in case the patient needed dialysis 7 days after transplantation (20). Acute rejection episodes were classified according to the updated Banff classification (21). Allograft loss was considered if eGFR dropped below 10 ml/min/1.73 m^2^ or if the patient returned to dialysis. All recipients were followed up during the entire first post-transplantation year unless death or graft loss occurred earlier. Red blood cell (RBC) transfusions were indicated according to hemoglobin level as follows (22):

- Hemoglobin < 6 g/dl: Transfusion is recommended except in exceptional circumstances;
- Hemoglobin 6–7 g/dl: Transfusion is likely to be indicated;
- Hemoglobin 7–8 g/dl: Transfusion may be appropriate in patients undergoing orthopedic or cardiac surgery, and in those with stable cardiovascular disease, after evaluating the patient’s clinical status;
- Hemoglobin 8–10 g/dl: Transfusion is generally not indicated but should be considered in some situations (e.g., symptomatic anemia, ongoing bleeding, acute coronary syndrome with ischemia); and
- Hemoglobin > 10 g/dl: Transfusion is generally not indicated except in exceptional circumstances.

Platelet transfusion was prescribed for therapeutic or prophylactic purposes upon reaching certain thresholds, i.e., 50,000 platelets/µl in active bleeding and in preoperative status for the former and 30,000 platelets/µl as prophylaxis against spontaneous bleeding (23).

### Immunosuppressive regimen

We defined two groups of patients according to induction therapy type: ATG-Thymoglobulin^®^ (Thymo group) and ATG-Fresenius (ATG-F group). Thymoglobulin^®^ (Sanofi-Aventis^®^, France) and ATG-F (renamed Grafalon^®^, Neovii^®^ Biotech GmbH, Switzerland) are the most commonly prescribed polyclonal antibodies in induction immunosuppressive protocols used in kidney transplantation (8). Patients received treatment on a one-in-two basis. Reasons for ATG induction were having a cPRA higher than 50% and/or preformed DSA and/or more than one solid organ transplantation. Patients in the Thymo group received 1.5 mg/kg daily, and those in the ATG-F group received 3 mg/kg of ATG-F, both for a period of 5 days after transplantation.

Considering maintenance immunosuppressive therapy, all patients received a combination of mycophenolate mofetil (MMF) 1,000 mg orally twice daily, tacrolimus 4 days after transplantation (0.15 mg/kg/day, trough level 7 to 9 ng/ml in the first 2 months, then 5 to 7 ng/ml), and steroids tapered over 3 months to 5 mg/day.

Infection-preventive strategy included sulfamethoxazole plus trimethoprim orally for at least 12 months as prophylaxis against *Pneumocystis carinii* pneumopathy (PCP), in addition to valganciclovir at a preventive dose for 3 to 6 months against CMV. Recipients who were seronegative for CMV and whose donor was also seronegative did not need valganciclovir.

### Anti-HLA antibody screening

Low-resolution DNA typing was performed in donors and high-resolution typing in recipients (HLA-A, HLA-B, Cw, HLA-DR, or HLA-DQ). Sera drawn before and after transplantation was examined for the presence of circulating preformed DSA and *de novo* DSA (dnDSA) directed against the donor’s HLA-A, HLA-B, HLA-Cw, HLA-DR, or HLA-DQ antigens, using high-resolution Luminex SAB assay technology (One Lambda, Inc., Canoga Park, CA, USA) on a Luminex platform. All beads showing a normalized MFI >500 were considered positive. For each serum sample, the concentrations of DSA and MFI max were reported.

### Primary and secondary endpoints

The primary endpoint was having an RBC transfusion within 14 days after transplantation. Secondary endpoints were as follows: (i) RBC transfusion within 28 days after transplantation, (ii) total volume of RBC transfusion, (iii) kidney allograft survival 12 months after transplantation, (iv) patient survival 12 months after transplantation, (v) eGFR 12 months after transplantation, (vi) acute rejection incidence 12 months after transplantation, and (vii) *de novo* DSA incidence at 12 months. Only the first episode of acute rejection was considered.

### Statistical methods

Continuous variables were expressed in mean (standard deviation (SD)) or median (interquartile range (IQR)) as appropriate. Categorical variables were expressed in *N* (%). According to variable distribution, we used the Student’s *t*-test or Wilcoxon test for continuous variables and the chi-square or Fisher’s exact tests for categorical variables. For the multivariable analyses, we used logistic regression.

A *p*-value of < 0.05 was considered significant. The tests were two-tailed. Analyses were performed using Stata v16.0 (StataCorp, College Station, TX, USA) and Prism 7 (GraphPad, USA).

## Results

**Figure 1:**
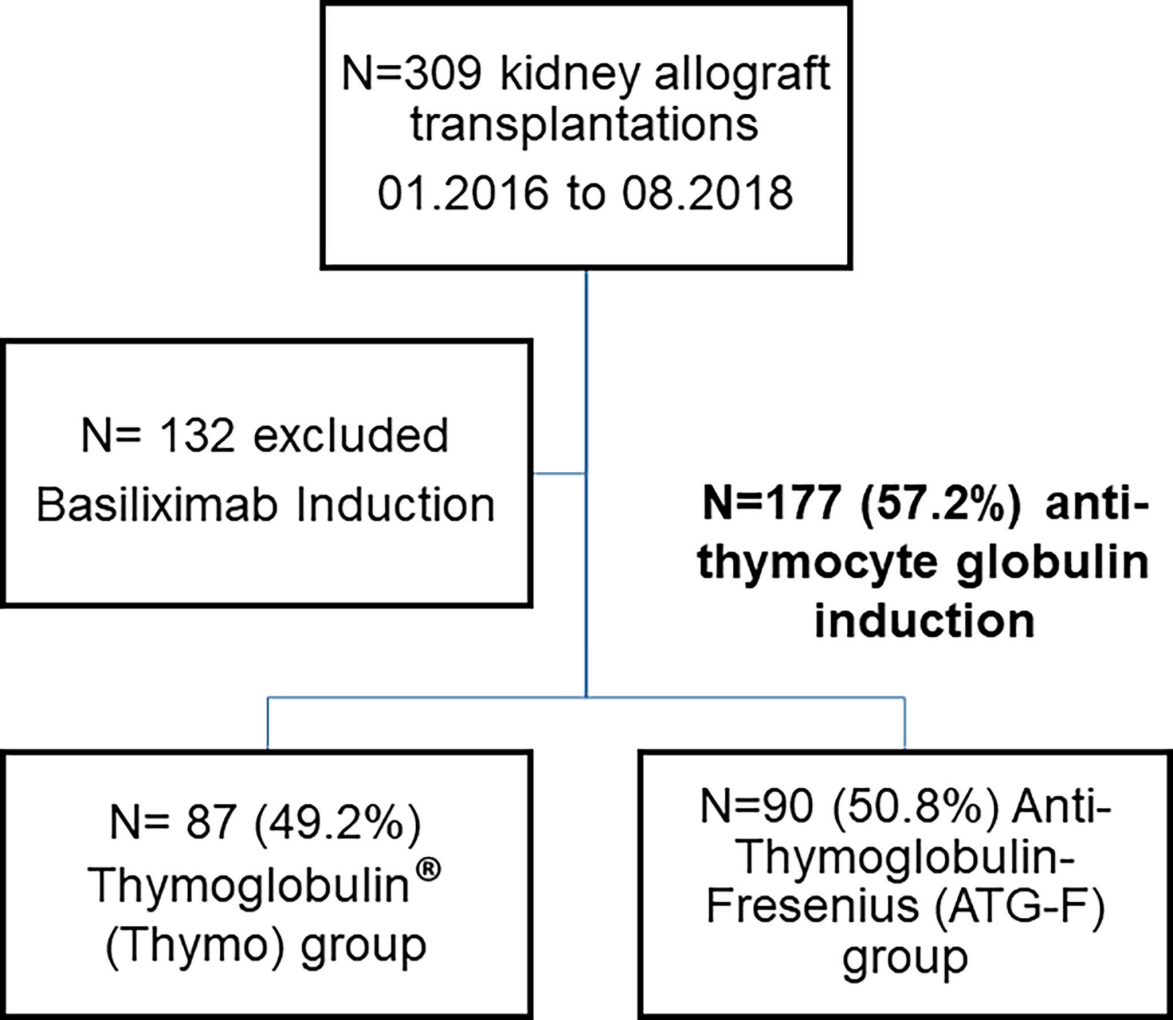
Among the 309 kidney allograft recipients engrafted between January 2016 and August 2018, 177 (57.2%) received anti-thymocyte globulin induction. *N* = 132 were excluded from the study because of basiliximab induction. ATG-F was given to 90 patients (50.8%), and Thymo was given to 87 (49.2%).

Of the 309 kidney allograft recipients, 177 (57.2%) received ATG induction. ATG-F was given to 90 patients (50.8%), and Thymo was given to 87 (49.2%) (**Figure 1**). At inclusion, the characteristics of recipients, their immunological risk before transplantation, donors, and of kidney transplantation were similar in both groups (**Table 1**). The use of anticoagulant and antiplatelet agents, before and at the time of transplantation, in both groups was also similar (**Table 1**). Similar to the maintenance immunosuppressive regimen, which included a combination of tacrolimus, MMF, and steroids in almost all patients (*p* = 0.73).

**Table 1:** Patients’ characteristics at the time of transplantation and their hematological parameters after transplantation.

	Thymo^®^ *n* = 87	ATG-F *n* = 90	*p*-value
**Recipient**			
Sex (male, *N* (%))	53 (61)	51 (57)	0.57
Age (years)	55 ± 14	56 ± 13	0.69
Body Mass Index (kg/m²; mean ± SD)	25 ± 3	26 ± 5	0.43
Initial nephropathy			
*Glomerulopathy (N (%))*	33 (37)	27 (30)	0.60
*Interstitial (N (%))*	1 (1)	5 (6)	
*Vascular (N (%))*	12 (14)	9 (10)	
*Genetic (N (%))*	12 (14)	16 (18)	
*Immunologic (N (%))*	4 (5)	4 (4)	
*Urologic (N (%))*	4 (5)	4 (4)	
*Undetermined (N (%))*	21 (24)	25 (28)	
Comorbidities			
*Hypertension (N (%))*	76 (87)	78 (87)	0.89
*Diabetes mellitus (N (%))*	21 (24)	18 (20)	0.50
*Sickle cell disease (N (%))*	4 (5)	5 (6)	0.77
Dialysis (*N* (%))	80 (92)	85 (94)	0.56
*Duration (months, median (IQR))*	43 (23–67)	45 (23–74)	0.91
**Donor**			
Age (years, mean ± SD)	57 ± 14	57 ± 15	0.88
Deceased donor (*N* (%))	73 (84)	77 (86)	0.85
eGFR (ml/min/1.73 m^2^; mean ± SD)	86 ± 30	86 ± 32	0.66
Extended criteria donor	49 (56)	46 (51)	0.54
**Immunological risk**			
Previous transplantation	10 (11)	20 (22)	0.07
Preformed anti-HLA donor-specific antibodies	43 (49)	42 (47)	0.76
Class I (*N* (%))	25 (29)	21 (23)	0.69
Class II (*N* (%))	29 (33)	30 (33)	
**Kidney transplantation**			
Cold ischemia time (h, median (IQR))	17 (13–23)	16 (13–21)	0.49
Maintenance therapy			
*Tacrolimus (N (%))*	77 (88)	85 (94)	0.73
*Mycophenolate mofetil (N (%))*	84 (96)	90 (100)	
*m-TOR inhibitors (N(%))*	4 (5)	4 (4)	
*Steroids (N (%))*	87 (100)	86 (95)	
*Belatacept de novo (N (%))*	10 (11)	5 (6)	
Delayed graft function (*N* (%))	25 (29)	29 (32)	0.62
**Treatment before and after transplantation**			
Curative anticoagulant (*N* (%))	14 (16)	14 (16)	0.92
Prophylactic anticoagulation (*N* (%))	79 (94)	77 (96)	0.51
Antiplatelet agents (*N* (%))	14 (47)	31 (34)	0.09
Erythropoiesis-stimulating agents (*N* (%))	43 (49)	59 (65)	0.03
**Hematologic parameters after transplantation**			
Within 14 days after transplantation			
Hemoglobin (g/dl, mean ± SD)	9.56 ± 1.22	8.62 ± 1.09	<0.01
Red blood cells transfusion (*N* (%))	40 (46)	57 (63)	0.02
Number of red blood cells units (median (IQR))	0 (0–2)	2 (0–4)	0.01
Platelets count (G/L, mean ± SD)	242 ± 89	262 ± 106	0.20
Platelets transfusion (*N* (%))	3 (3)	5 (6)	0.50
Within 28 days after the transplant			
Hemoglobin (g/dl, median (IQR))	10.62 ± 1.34	9.79 ± 1.31	<0.01
Red blood cells transfusion (*N* (%))	45 (52)	59 (66)	0.06
Number of red blood cells units (median (IQR))	0 (0–2)	2 (0–4)	0.04
Platelet count (G/L, mean ± SD)	225 ± 79	217 ± 90	0.52
Platelet transfusion (*N* (%))	3 (3)	5 (6)	0.50

**Figure 2:**
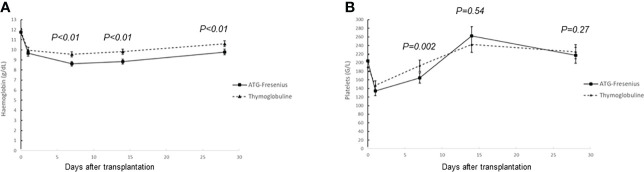
Hemoglobin concentration and platelet count within the first month after kidney transplantation. **(A)** Hemoglobin concentration: the hemoglobin concentrations were significantly lower at days 7, 14, and 28 in the ATG-F group, as compared with the Thymo group (*p* < 0.01 for all points). **(B)** Platelet count: the platelet count was significantly lower in the ATG-F group at day 7 (193 ± 64 G/L in the Thymo group vs. 164 ± 58 G/L in the ATG-F group; *p* = 0.003) but similar at days 14 and 28 (*p* = 0.54 and *p* = 0.27, respectively) in both groups.

Hemoglobin concentration was significantly lower on days 7, 14, and 28 in the ATG-F group (**Figure 2A**; **Table 1**). The ATG-F group had significantly lower platelet count than the Thymo group at day 7 (164 ± 58 vs. 193 ± 64 G/L; *p* = 0.003) but similar at days 14 and 28 (*p* = 0.54 and *p* = 0.27, respectively) in both groups (**Figure 2B**; **Table 1**). Fifty-seven patients (63.3%) of the ATG-F group required red blood cell transfusion in the first 14 days after kidney transplantation, compared with 40 patients (46%) of the Thymo group (*p* = 0.02). The volume of RBC transfusion was significantly higher in the ATG-F group (**Table 1**, *p* = 0.01). Platelet transfusion was similar in both groups and concerned *N* = 3 (3%) patients of the Thymo group and *N* = 5 (6%) patients of the ATG-F group (**Table 1**).

We observed significantly more surgical complications within 1 month after transplantation (e.g., hematomas, urinoma, delayed wound healing), in the Thymo group than in the ATG-F group (*N* = 32 (36.8%) vs. *N* = 20 (22.2%), respectively; *p* = 0.03). Hemorrhage was similar in both groups (23% vs. 16%, respectively; *p* = 0.29).

Analysis at day 14 after kidney transplantation showed that older age, the ATG-F induction regimen, anticoagulant use, and early surgical complications were associated with RBC transfusion through that period, whereas at day 30, only older age and early surgical complications were associated with RBC transfusion (**Table 2**). Within 14 and 30 days after transplantation, older age, induction with ATG-F, and early surgical complications were independently associated with RBC transfusion (**Table 2**). After adjustment with hemoglobin before transplantation (day 0), after transplantation (day 1), or both, the ATG-F induction regimen remains independently associated with RBC transfusion (**Supplementary Table S1**).

**Table 2:** Risk factors for red blood cell transfusion.

	Univariable analysis		Multivariable analysis	
	OR	*p*	OR	*p*
**Within 14 days**				
Age	1.05 [1.02–1.07]	< 0.01	1.04 [1.01–1.07]	< 0.01
Sex (female)	0.75 [0.41–1.38]	0.36	0.76 [0.39–1.48]	0.43
Anticoagulation	2.88 [1.16–7.19]	0.02	2.05 [0.74–5.73]	0.17
Early surgical complication	3.47 [1.69–7.19]	< 0.01	3.59 [1.60–8.03]	< 0.01
Anti-thymocyte serum, ATG-F	2.03 [1.11–3.70]	0.02	2.85 [1.43–5.68]	< 0.01
**Within 30 days**				
Age	1.04 [1.02–1.07]	< 0.01	1.04 (1.01–1.06]	< 0.01
Sex (female)	0.83 [0.45–1.53]	0.56	0.87 (0.45–1.67]	0.67
Anticoagulation	2.39 [096–5.95]	0.06	1.65 (0.6–4.49]	0.33
Early surgical complication	3.18 [1.52–6.62]	< 0.01	3.11 (1.39–6.92]	< 0.01
Antithymocyte serum, ATG-F	1.78 [0.97–3.2]	0.06	2.28 (1.17–4.46]	0.02

Patients were followed up for 12 months after kidney transplantation, as shown in **Table 3**. One patient was lost to follow-up. Patient survival rate was 95% (*N* = 168/176), and the death-censored kidney allograft survival rate was 92% (*N* = 162/176). Causes of death were infections (*N* = 5), cardiorespiratory arrest (*N* = 1), neoplasia (*N* = 1), and unknown (*N* = 1). Causes of kidney allograft failure were arterial thrombosis (*N* = 3), acute rejection (*N* = 3), and infection (*N* = 1). Patient and kidney allograft survivals, as well as the 12-month eGFR, were similar in both groups (**Table 3**). The incidence of acute rejection was 20% (*N* = 17) in the Thymo group and 13% (*N* = 12) in the ATG-F group (*p* = 0.49). The time to develop acute rejection was similar in both groups. Infections, including CMV, happened in both groups at similar rates. The percentage of acute rejection in the first year after transplantation was similar in patients who required RBC transfusion and those who did not (*N* = 18/104 (17%) vs. *N* = 10/73 (14%); *p* = 0.68).

**Table 3:** Patients’ follow-up at 12 months.

	Thymo^®^	ATG-F	*p*-value
	*n* = 87	*n* = 90	
**12-Month kidney transplantation evolution**			
Recipient death (*N* (%))	4 (5)	4 (4)	1.00
Kidney allograft loss (*N* (%))	3 (3)	4 (4)	1.00
Functional kidney allograft (*N* (%))	80 (92)	81 (90)	1.00
eGFR (ml/min/1.73 m², mean ± SD)	49.22 ± 20.93	48.31 ± 20.28	0.78
Proteinuria (mg/mmol; median (IQR))	16 (8.1–33)	16.45 (8.76–34.42)	0.10
**Acute rejection** (*N* (%))	17 (20)	12 (13)	0.49
T-cell–mediated rejection (*N* (%))	9 (10)	9 (10)	0.47
Antibody-mediated rejection (*N* (%))	6 (7)	2 (2)	
Mixed rejection (*N* (%))	2 (2)	1 (1)	
Time to rejection (months, median (IQR))	3 (0.25–6.25)	3 (2.25–9.5)	0.45
**Anti-HLA donor-specific antibodies (*N* (%))**	36/80 (45)	36/90 (44)	0.54
Class I (*N* (%))	10/36 (28)	20/36 (56)	0.08
Class II (*N* (%))	34/36 (94)	25/36 (61)	
**Complications**			
New onset diabetes (*N* (%))	22 (25)	16 (18)	0.22
Infection (*N* (%))	29 (33)	29 (32)	0.13
Bacterial (*N* (%))	41 (47)	43 (48)	0.73
Viral (*N* (%))	47 (54)	40 (44)	0.53
BK virus viremia (*N* (%))	11 (13)	13 (14)	0.35
BK nephropathy (*N* (%))	1 (1)	4 (4)	0.19
CMV viremia (*N* (%))	18 (21)	23 (26)	0.69
Fungal infection (*N* (%))	10 (11)	10 (11)	0.31

Previous transplantation and the percentage of preformed DSA at the time of transplantation were similar in both groups (**Table 1**). The prevalence of DSA within the 12 months after transplantation was similar irrespective of induction regimen or whether recipients required RBC transfusion or not (*N* = 37/104 (36%) vs. *N* = 35/73 (48%); *p* = 0.12).

## Discussion

Herein, we present the results of our monocentric observational study comparing the need for transfusion after kidney transplantation in patients receiving either an ATG-F or Thymo induction regimen. Upon comparing both ATGs associated with the same immunosuppressive maintenance regimen, ATG-F is an independent risk factor for RBC transfusion up to 14 days after kidney transplantation. Although ATG-F was associated with thrombocytopenia in the first month after kidney transplantation, it did not give rise to more platelet transfusions. We did not notice any clinical or biological effects of the increased RBC transfusion rate, i.e., acute rejection rate and/or DSA prevalence in the 12 months that followed kidney transplantation were similar in both groups. We also reported similar efficacy of ATG-F and Thymo in preventing acute rejection and delayed graft function, as well as improving patient and kidney allograft survivals in sensitized recipients.

Our results showed a higher incidence of anemia and RBC transfusions in the ATG-F group. Anemia has already been reported as a side effect of ATG-F rather than Thymo induction (10) after kidney transplantation, but the need for RBC transfusion unequivocally depends on the ATG regimen (9, 10, 12). RBC transfusion is widely considered a risk factor for sensitization before and after kidney transplantation, with a higher incidence of DSA development in the first year after transplantation, leading to a higher incidence of acute antibody-mediated rejection (ABMR) in the RBC-transfused patients (24, 25). Recently, two reports did not find an association between RBC transfusion and acute rejection (26, 27). Some authors suggested that RBC transfusions could be given safely to kidney-transplant recipients in the early post-transplantation period (first 3 months) provided that they receive an ATG-based induction therapy (27). In addition to the significantly higher anemia incidence and RBC transfusion rate in the ATG-F group, we reported no increase in the incidence of DSA in RBC-transfused cases in the 12 months that followed kidney transplantation. Acute rejection, whether ABMR or T-cell mediated, had a similar incidence in the year following kidney transplantation.

ATG-F has already been implicated as a cause of early thrombocytopenia after kidney transplantation, particularly when associated with everolimus (10–13, 28, 29). Some studies reported that the lowest platelet count was reached on day 3 (12, 30). Clinically speaking, clinicians would rarely let platelet count decrease below 50 G/L without ordering frequent platelet transfusions (13, 28); however, no such clinical outcomes have been reported (13). Our study confirmed early, not severe thrombocytopenia occurring within 7 days after kidney transplantation that did not need frequent platelet transfusion. We also confirmed the absence of clinical consequences, especially hemorrhage and surgical complications.

Patient and kidney allograft survivals within the first year did not differ between ATG induction types. A meta-analysis documented the inferiority of Thymo to ATG-F in preventing delayed graft function, patient death, and graft loss (8). However, in studies directly comparing Thymo with ATG-F, no difference in terms of DGF and patient and allograft survivals was isolated (8). Recently, a large cohort of adult kidney transplant recipients has shown that transfusing RBC after transplantation may increase the risk for death-censored graft loss (26). Given the low number of RBC-transfused patients in our study, we could not analyze the risk for patient and kidney allograft survival. Such parameters merit to be investigated in other cohorts.

Finally, we analyzed drug safety in the first year after kidney transplantation. We did not detect more infections, especially CMV, in the Thymo group or *de novo* diabetes after transplantation, as previously described (7, 8, 28). In simultaneous pancreas–kidney transplantation, infections did not happen more in the ATG-F group, compared with the Thymo group (11). A short follow-up period after our study did not allow us to analyze long-term drug safety.

ATG-F and Thymo seemed to be equivalent in terms of their efficacy in preventing acute rejection after kidney transplantation in high immunological-risk recipients. However, hematological toxicity on red blood cells and platelets is more common in ATG-F–treated patients. The mechanisms of anemia and thrombocytopenia after ATG treatment remained unclear. Some authors described links between complement activation and thrombin generation, and hypercoagulability and thrombocytopenia (31). A higher degree of vascular inflammation in kidney transplant recipients might increase platelets’ response to ATG, thus increasing the risk of thrombocytopenia. Complement activation could favor anemia. Although ATG-F and Thymo are both rabbit-derived antibodies, their production relies on different methods, which could explain differences in secondary effects after infusion. ATG-F comprises only rabbit antibodies formed upon exposure to the Jurkat cell line (6), whereas Thymoglobulin is a pasteurized antibody produced by immunizing rabbits with fresh human thymocytes (6).

In highly sensitized living donor recipients, alternatives to ATG have been explored. The association of rituximab, plasma exchanges, and donor-specific immunosuppression could lead to better results than ATG and lower secondary effects (5). In patients with one detection of DSA with MFI lower than 1,000, ATG used should be discussed as the interassay variability is high (32). However, these treatments are not easy to use in deceased donor kidney allograft recipients, as cold ischemia time is an independent factor of allograft survival (33).

We recognized certain limitations in our study. The incidence of DSA in up to 50% of our cohort is high, probably because of the threshold of 500 MFI considered. However, it is the first to compare, in similar groups of kidney allograft recipients, safety, especially hematological safety, and efficacy, including DSA data over 12 months after kidney transplantation.

To conclude, our study confirmed that, when combined with maintenance therapy with calcineurin inhibitors, mycophenolate mofetil, and steroids, ATG-F after kidney transplantation is an independent risk factor for early RBC transfusion (in the first month) and early thrombocytopenia without clinical (patient and kidney allograft survivals, acute rejection, infections) and biological effects, especially the development of dnDSA. The use of ATG-F or Thymo after kidney transplantation has to be balanced considering these new data, and alternatives to ATG use in sensitized kidney allograft recipients’ merit further exploration.

## Data availability statement

The raw data supporting the conclusions of this article will be made available by the authors, without undue reservation.

## Ethics statement

The studies involving human participants were reviewed and approved by CPP IRB #00003835. Written informed consent for participation was not required for this study in accordance with the national legislation and the institutional requirements.

## Author contributions

MS and CP-H equally contributed to this work and share first authorship. MS, CP-H, CO,CC-J, MP, PG and MM contributed to the conception and design of the study. MS, CP-H, BC and MM collected the data. MS, CP-H, ÉA, CO, and MM contributed to analysis and interpretation of data. MS, CP-H, and MM drafted the article and all other authors revised it critically for important intellectual content. All authors approved the final version of the manuscript.

## Acknowledgments

We thank Tiphanie Londero who extracted all data from our database.

## Conflict of interest

The authors declare that the research was conducted in the absence of any commercial or financial relationships that could be construed as a potential conflict of interest.

## Publisher’s note

All claims expressed in this article are solely those of the authors and do not necessarily represent those of their affiliated organizations, or those of the publisher, the editors and the reviewers. Any product that may be evaluated in this article, or claim that may be made by its manufacturer, is not guaranteed or endorsed by the publisher.

## Abbreviations

ABMR, acute antibody-mediated rejection; ATG, anti-thymocyte globulins; ATG-F, anti-thymocyte globulin-Fresenius; BMI, body mass index; CNI, calcineurin inhibitors; cPRA, calculated panel reactive antibodies; dnDSA, *de novo* donor-specific antibodies; DSA, donor-specific antibodies; DGF, delayed graft function; ECD, extended criteria donor; ESRD, end-stage renal disease; eGFR, estimated glomerular filtration rate; HLA, human leucocyte antigen; MMF, mycophenolate mofetil; PCP, *Pneumocystis carinii* pneumopathy; RBC, red blood cells; TCMR, T-cell–mediated rejection; Thymo, Thymoglobulin^®^.
