# Supplementary material for: ATG-Fresenius increases the risk of red blood cell transfusion after kidney transplantation
Source: Front Immunol. 2022 Dec 1;13:1045580. doi: 10.3389/fimmu.2022.1045580 (PMC9753326; doi:10.3389/fimmu.2022.1045580)
Supplement: Supplementary file 1 [file Table_1.docx]

Supplementary table 1: Risk factors for red blood cell transfusion with adjustment on haemoglobin level

|  | **Multivariable analysis** | | |
| --- | --- | --- | --- |
|  | **OR** | ***p*** | |
| **Within 14 Days – Adjustment on day 0 haemoglobin** |  | |  |
| Haemoglobin day 0 | 0.75 [0.59 – 0.95] | | 0.02 |
| Age | 1.04 [1.02 – 1.07] | | < 0.01 |
| Sex, female | 0.70 [0.35 – 1.38] | | 0.30 |
| Anticoagulation | 2.15 [0.77 – 6.01] | | 0.15 |
| Early surgical complication | 3.64 [1.61 – 8.24] | | < 0.01 |
| Anti-thymocyte serum, ATG-F | **2.91 [1.43 – 5.89]** | | **< 0.01** |
|  |  | |  |
| **Within 14 Days – Adjustment on day 1 haemoglobin** |  | |  |
| Haemoglobin day 1 | 0.53 [0.40 – 0.71] | | < 0.01 |
| Age | 1.05 [1.02 – 1.08] | | < 0.01 |
| Sex, female | 0.71 [0.34 – 1.49] | | 0.37 |
| Anticoagulation | 2.66 [0.85 – 8.27] | | 0.09 |
| Early surgical complication | 3.11 [1.39 – 6.92] | | < 0.01 |
| Anti-thymocyte serum, ATG-F | **2.68 [1.25 – 5.74]** | | **0.01** |
|  |  | |  |
| **Within 14 Days – Adjustment on day 0 and day 1 haemoglobin** |  | |  |
| Haemoglobin day 0 | 1.08 [0.80 – 1.46] | | 0.62 |
| Haemoglobin day 1 | 0.51 [0.36 – 0.72] | | < 0.01 |
| Age | 1.05 [1.02 – 1.08] | | < 0.01 |
| Sex, female | 0.73 [0.35 – 1.52] | | 0.39 |
| Anticoagulation | 2.61 [0.83 – 8.21] | | 0.10 |
| Early surgical complication | 3.46 [1.40 – 8.55] | | < 0.01 |
| Anti-thymocyte serum, ATG-F | **2.66 [1.24 – 5.72]** | | **0.01** |
